# Deletion of the *N*‐ and C‐termini of tau initiates its aggregation and acquires proteopathic properties

**DOI:** 10.1002/alz70855_107182

**Published:** 2025-12-25

**Authors:** Fei Liu, Ruozhen Wu, Dandan Chu, Jianlan Gu, Nana Jin, Yunn Chyn Tung, Allegra Sieh Ho, Zhihao Hu, Xiaochuan Wang, Khalid Iqbal

**Affiliations:** ^1^ New York State Institute for Basic Research in Developmental Disabilities, Staten Island, NY, USA; ^2^ Tongji Medical College, Huazhong University of Science and Technology, Wuhan, Hubei, China; ^3^ Nantong University, Nantong, Jiangsu, China

## Abstract

**Background:**

Intracellular neurofibrillary tangles (NFTs) consisting of aggregated and hyperphosphorylated tau is a major pathological hallmark of Alzheimer's disease and related tauopathies. In AD brain, tau pathology spreads through prion‐like propagation and follows a stereotypical pattern: it originates from the locus coeruleus and transentorhinal cortex (Braak stages I–II), then spreads to the limbic system (Braak stages III–IV), and eventually to the neocortex (Braak stages V–VI). Normally, the acidic *N*‐ and C‐termini of tau prefers to fold over the basic microtubule binding repeats (MTBR) to form a paperclip‐like conformation, preventing the protein from self‐aggregation. In AD brain, the extreme *N*‐terminus of tau is lost from NFTs and C‐terminal truncations are found in PHFs as well as mature and ghost tangles. Loss of the *N*‐ and C‐termini of tau may facilitate self‐aggregation and drive tau pathogenesis.

**Method:**

A truncated form of tau, tau_151‐391_ was generated by deleting the acidic *N*‐ and C‐termini of tau and overexpressed in HEK‐293T cells. RIPA buffer insoluble tau_151‐391_ aggregates were obtained by ultracentrifugation. Its phosphorylation and proteopathic properties were determined *in vitro* and *in vivo*.

**Result:**

We found that tau_151‐391_, but not full‐length tau (tau_FL_), can form aggregates in cultured cells and in vivo in mouse brain. Aggregated tau_151‐391_ is hyperphosphorylated at multiple sites, including Thr181, Ser199, AT8, Thr212, Ser214, Thr217, and Thr231, and is partially resistant to hydrolysis by proteinase K. Tau_151‐391_ aggregates potently induce tau aggregation and site‐specific hyperphosphorylation in cultured cells and in mouse brain. By repeatedly inducing aggregation in cultured cells, aggregated tau increases exponentially. The seeding activity of tau_151‐391_ aggregates is sensitive to repeated freezing and thawing. 3R‐tau_151‐391_, which contains three MTBRs, and 4R‐tau_151‐391_, which contains four MTBRs, can be induced to aggregate by each other's aggregates.

**Conclusion:**

Deletion of the acidic *N*‐ and C‐termini facilitates tau aggregation and acquires proteopathic properties. Tau_151‐391_ aggregates effectively induce tau aggregation and site‐specific hyperphosphorylation in cultured cells and *in vivo* in mouse brain.

